# (*E*)-2,6-Di­bromo-4-{2-[1-(1*H*,1*H*,2*H*,2*H*-perfluoro­oct­yl)pyridinium-4-yl]ethen­yl}phenolate methanol disolvate, a fluoro­ponytailed solvatochromic dye

**DOI:** 10.1107/S2056989017013378

**Published:** 2017-09-25

**Authors:** Lukas Fliri, Gabriel Partl, Thomas Gelbrich, Volker Kahlenberg, Gerhard Laus, Herwig Schottenberger

**Affiliations:** a University of Innsbruck, Faculty of Chemistry and Pharmacy, Innrain 80-82, 6020 Innsbruck, Austria; b University of Helsinki, Department of Chemistry, PO Box 55, 00014 Helsinki, Finland; c University of Innsbruck, Institute of Mineralogy and Petrography, Innrain 52, 6020 Innsbruck, Austria

**Keywords:** crystal structure, fluoro­alk­yl, methanol, phenolate, pyridinium, solvatochromism

## Abstract

The title compound, C_21_H_12_Br_2_F_13_NO·2CH_3_OH, was obtained by condensation of 4-methyl-1-(1*H*,1*H*,2*H*,2*H*-perfluoro­oct­yl)pyridinium iodide and 3,5-di­bromo-4-hy­droxy­benzaldehyde, followed by deprotonation. It crystallizes as a methanol disolvate and exhibits short O—H⋯O hydrogen bonds and a disordered perfluoro­alkyl chain [occupancy ratio 0.538 (7):0.462 (7)]. Significant π–π stacking inter­actions are observed between the benzene and pyridine rings of neighbouring mol­ecules along the *b-*axis direction.

## Abstract 2

The title compound crystallizes as a methanol disolvate and exhibits short hydrogen bonds and a disordered perfluoro­alkyl chain.

## Chemical context   

Dyes with a fundamental type of conjugated system as in the title compound have long been known (Hünig & Rosenthal, 1955 ▸). It was intended to combine the structural features of a delocalized π-electron system with those of polyfluorinated compounds in order to derive a new material with advantageous properties such as altered solubility (Hoang & Mecozzi, 2004 ▸) and affinity (Wagner *et al.*, 2016 ▸) profiles, given that the physical and chemical properties of organic compounds are strongly affected by the introduction of fluorinated substituents. Fluoro­surfactants have a tendency towards micelle formation in biphasic or ternary solvent mixtures. Thus, the utilization of solvatochromic surfactants as self-indicating micelle reporters (Kedia *et al.*, 2014 ▸) is an attractive analytical concept for fluorous-phase-related materials science.
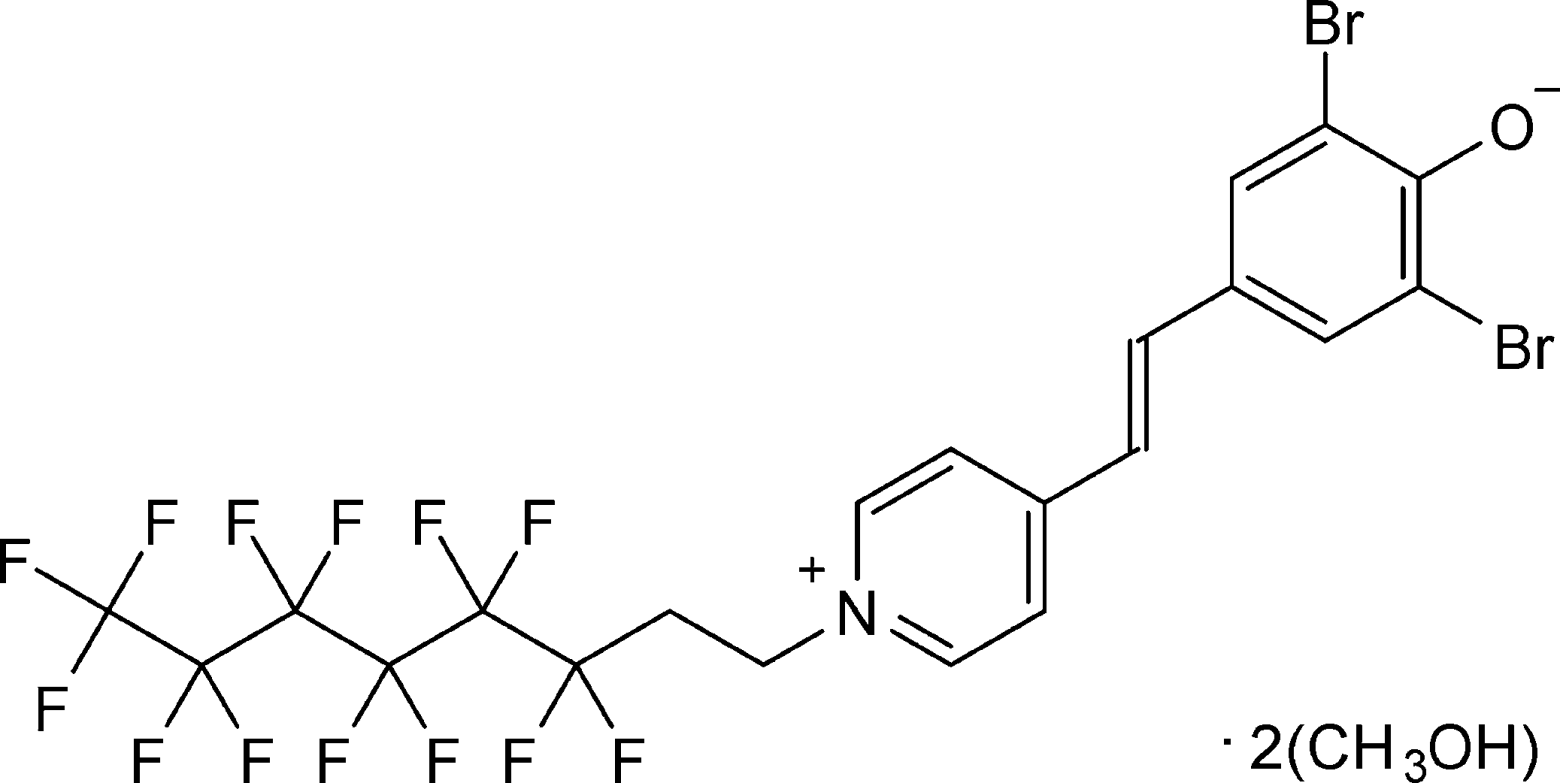


## Structural commentary   

The title compound comprises a delocalized π-electron system, involving either a zwitterionic benzoid or a non-polar quinoid resonance structure. Inspection of bond lengths leads to the conclusion that it is not a typical cyclo­hexa­dienone system (Chandran *et al.*, 2008 ▸; Chiverton *et al.*, 1991 ▸) but rather a benzoid system similar to 2,6-di­bromo­phenol predominant (Eriksson & Eriksson, 2001 ▸; Lu *et al.*, 2011 ▸; Lehmler & Parkin, 2005 ▸). The heterocyclic ring also resembles a typical pyridinium system. Furthermore, the shortest C=C bond in the bridge linking the two rings is between C6 and C7 with a length of 1.337 (6) Å, whereas the adjacent bonds are considerably longer. The framework thus is not quinoid but benzoid. The conjugated moieties of the dye mol­ecule are almost planar and the mean planes of the benzene and pyridine rings form an angle of 2.97 (2)°, whilst the fluorinated chains protrude from the plane.

The carbon atoms C17–C21 and fluorine atoms F3–F13 of the polyfluorinated tail are disordered over sets of sites with an occupancy ratio for the two disorder fragments of 0.538 (7):0.462 (7). The chain adopts a slightly helical conformation (Fournier *et al.*, 2010 ▸) with an average C—C—C—C twist angle (deviation from 180°) of 3°. Typically, π-electron donor–acceptor-substituted conjugated systems exhibit solvatochromism. Solutions of the title compound display absorption maxima at 610 nm (blue) in THF and 502 nm (red) in MeOH. Here, increased solvent polarity leads to higher transition energy (negative solvatochromism). A quinoid system based on 2,6-di­bromo­phenol displaying positive solvatochromism has been reported previously (Laus *et al.*, 2003 ▸).

## Supra­molecular features   

**Figure 1:**
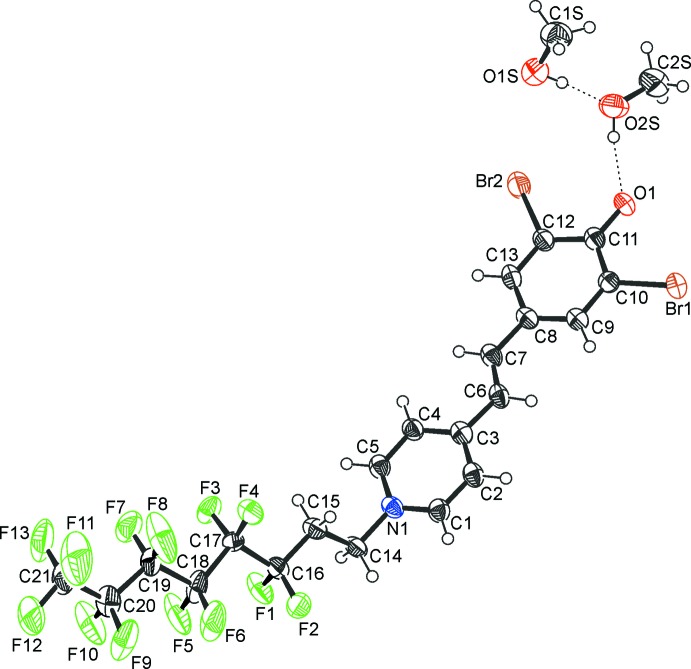
The mol­ecular structure of the title compound, with atom labels and 50% probability displacement ellipsoids for non-H atoms. Dashed lines indicate hydrogen bonds. Only the major disorder component of the perfluoro­alkyl chain is shown.

**Figure 2:**
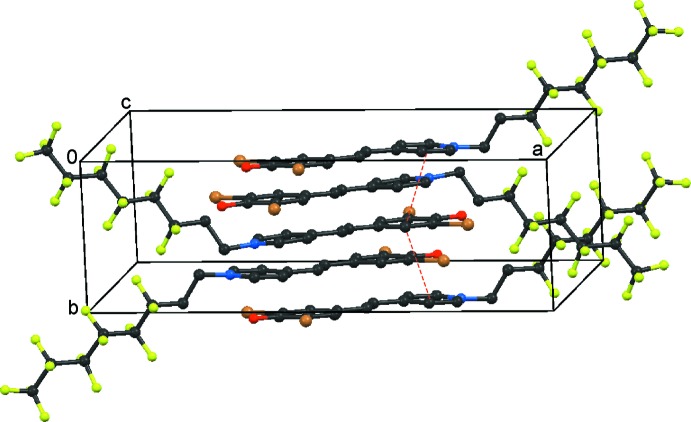
View of the planar chromophore moieties and the attached perfluoro­alkyl chains. The π–π stacking inter­actions between the benzene and pyridine rings are shown in red dashed lines. Solvent mol­ecules are omitted for clarity.

The three components of the title compound are linked into a finite hydrogen-bonded chain. The two solvent mol­ecules are connected by an O1*S*—H1*S*⋯O2*S* bond, and additionally the inter­action O2*S*—H2*S*⋯O1 links the second solvent mol­ecule with the main mol­ecule (Table 1 ▸, Fig. 1 ▸). In addition, there are significant π–π stacking inter­actions between the benzene and pyridine rings. These are weakly connecting in the *b-*axis direction. Centroid–centroid distances *Cg*1⋯*Cg*2^i^ and *Cg*1⋯*Cg*2^ii^ are 3.525 (3) and 3.605 (3) Å, respectively [*Cg*1 and *Cg*2 are the centroids of the benzene and pyridine rings, respectively; symmetry codes: (i) 1 − *x*, −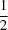 + *y*, 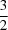 − *z*; (ii) 1 − *x*, 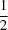 + *y*, 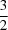 − *z*]. The packing of the mol­ecules is displayed in Fig. 2 ▸.

## Database survey   

The crystal structure of an acceptor-substituted conjugated 2,6-di­bromo­phenol derivative (refcode SULSAV), displaying visible solvatochromism, has been reported (Stock *et al.*, 2015 ▸).

## Synthesis and crystallization   

**4-Methyl-1-(1** ***H*** **,1** ***H*** **,2** ***H*** **,2** ***H*** **- perfluoro­oct­yl)pyridinium iodide (1):** A solution of 4-methyl­pyridine (10.0 g, 107.4 mmol) and 1*H*,1*H*,2*H*,2*H*-perfluoro­octyl iodide (66.2 g, 139.2 mmol) in CH_3_CN (15 ml) was refluxed for 24 h. The mixture was diluted with Et_2_O (250 ml) and allowed to rest at 249 K overnight. The product **1** was collected by filtration, washed with Et_2_O (100 ml) and dried to give 59.3 g (97%) of a dark-red powder. ^1^H NMR (300 MHz, CD_3_OD): δ 8.97 (*d*, *J* = 6.5 Hz, 2H), 8.01 (*d*, *J* = 6.5 Hz, 2H), 5.01 (*t*, *J* = 7.2 Hz, 2H), 3.25–3.04 (*m*, 2H), 2.71 (*s*, 3H) ppm.

**(** ***E*** **)-4-(2-(3,5-Di­bromo-4-hy­droxy­phen­yl)ethen­yl)-1-(1** ***H*** **,1** ***H*** **,2** ***H*** **,2** ***H*** **-perfluoro­oct­yl)pyridinium iodide (2):** A solution of inter­mediate **1** (2.03 g, 3.57 mmol), 3,5-di­bromo-4-hy­droxy­benzaldehyde (1.00 g, 3.57 mmol) and piperidine (0.5 ml, 5 mmol) in MeOH (10 ml) was refluxed for 4 h. After removal of the solvent, the residue was washed with CHCl_3_ (50 ml) and H_2_O (20 ml), dissolved in MeOH (70 ml) and precipitated with Et_2_O (400 ml). The crude product (2.0 g) was redissolved in acetone (40 ml) and precipitated with H_2_O (400 ml), filtered and dried to give 1.53 g (52%) of **2** as a red–brown powder, m.p. 497 K. ^1^H NMR (300 MHz, CD_3_OD): δ 8.52 (*d*, *J* = 6.7 Hz, 2H), 7.81 (*d*, *J* = 6.6 Hz, 2H), 7.73 (*s*, 2H), 7.63 (*d*, *J* = 15.8 Hz, 1H), 6.82 (*d*, *J* = 15.8 Hz, 1H), 4.78 (*t*, *J* = 7.2 Hz, 2H), 3.18–2.93 (*m*, 2H) ppm IR (neat): ν 3035(*w*), 3002(*w*), 2956(*w*), 1641(*w*), 1601(*m*), 1563(*m*), 1499(*m*), 1469(*m*), 1425(*w*), 1366(*w*), 1315(*w*), 1232(*m*), 1171(*s*), 1140(*vs*), 1075(*m*), 1041(*m*), 958(*m*), 916(*w*), 859(*m*), 809(*w*), 780(*w*), 745(*m*), 717(*m*), 695(*m*), 652(*m*), 616(*m*), 588(*m*), 551(*w*), 514(*m*), 488(*w*) cm^−1^.

**(** ***E*** **)-2,6-Di­bromo-4-(2-(1-(1** ***H*** **,1** ***H*** **,2** ***H*** **,2** ***H*** **-perfluoro­oct­yl)pyridinium-4-yl)ethen­yl)phenolate methanol disolvate (3):** A solution of NaOH (2 ml 5%, 2.5 mmol) was added to inter­mediate **2** (1.0 g, 1.2 mmol) in MeOH (20 ml). The mixture was ultrasonicated for 25 min, then heated and diluted with H_2_O (400 ml). After resting at 277 K overnight, the mixture was filtered and the dark-red product **3** was collected and dried: 0.57 g (68%). M.p. 513 K. Suitable crystals were obtained by diffusion of Et_2_O into a solution of **3** in MeOH at 249 K. ^1^H NMR (300 MHz, CD_3_OD): δ 8.52 (*d*, *J* = 6.3 Hz, 2H), 7.81 (*d*, *J* = 6.3 Hz, 2H), 7.73 (*s*, 2H), 7.63 (*d*, *J* = 15.8 Hz, 1H), 6.90–6.75 (*m*, 1H), 4.78 (*t*, *J* = 7.2 Hz, 2H), 3.17–2.95 (*m*, 2H) ppm IR (neat): ν 3642(*w*), 3305(*w*), 3037(*w*), 2999(*w*), 2958(*w*), 2939(*w*), 1641(*m*), 1601(*m*), 1562(*s*), 1523(*s*), 1499(*s*), 1469(*m*), 1425(*m*), 1366(*w*), 1315(*w*), 1231(*m*), 1171(*vs*), 1140(*vs*), 1119(*s*), 1076(*m*), 1041(*m*), 996(*w*), 959(*m*), 916(*w*), 857(*m*), 809(*m*), 780(*w*), 745(*m*), 717(*s*), 707(*m*), 694(*s*), 653(*m*), 616(*m*), 589(*m*), 565(*w*), 551(*m*), 530(*m*), 516(*m*), 491(*w*) cm^−1^.

## Refinement   

Crystal data, data collection and structure refinement details are summarized in Table 2 ▸. All H atoms were identified in difference maps. Methyl H atoms were idealized and included as rigid groups allowed to rotate but not tip and refined with *U*_iso_(H) set to 1.5*U*_eq_(C) of the parent carbon atom. All other H atoms bonded to carbon atoms were positioned geometrically and refined with *U*_iso_(H) set to 1.2*U*_eq_(C) of the parent carbon atom. Hydrogen atoms in OH groups were refined with restrained distances [O—H = 0.84 (1) Å] and their *U*_iso_ parameters were refined freely.

The terminal C_5_F_11_ unit of the polyfluorinated tail was found to be disordered over two orientations. The two disorder components, each consisting of 16 atomic positions, were refined using 401 distance restraints (SADI) for chemically equivalent C—C, C—F and F⋯F bonds, and the final occupancy ratio was 0.538 (7):0.462 (7). All disordered atoms were refined anisotropically. The extension of the modelled disorder increased the number of refined parameters substanti­ally. Consequently, the obtained data/parameter ratio is lower than normally expected.

## Supplementary Material

Crystal structure: contains datablock(s) I, global. DOI: 10.1107/S2056989017013378/lh5850sup1.cif

Structure factors: contains datablock(s) I. DOI: 10.1107/S2056989017013378/lh5850Isup2.hkl

Supporting information file. DOI: 10.1107/S2056989017013378/lh5850Isup3.mol

Supporting information file. DOI: 10.1107/S2056989017013378/lh5850Isup4.cml

CCDC reference: 1575370

Additional supporting information:  crystallographic information; 3D view; checkCIF report

## Figures and Tables

**Table 1:** Hydrogen-bond geometry (Å, °)

*D*—H⋯*A*	*D*—H	H⋯*A*	*D*⋯*A*	*D*—H⋯*A*
O1*S*—H1*S*⋯O2*S*	0.85 (1)	1.83 (1)	2.674 (5)	176 (7)
O2*S*—H2*S*⋯O1	0.84 (1)	1.85 (2)	2.675 (4)	167 (6)

**Table 2:** Experimental details

Crystal data	
Chemical formula	C_21_H_12_Br_2_F_13_NO·2CH_4_O
*M*_r_	765.22
Crystal system, space group	Monoclinic, *P*2_1_/*c*
Temperature (K)	173
*a*, *b*, *c* (Å)	22.2362 (7), 6.7922 (18), 18.9098 (5)
β (°)	103.989 (3)
*V* (Å^3^)	2771.3 (7)
*Z*	4
Radiation type	Cu *K*α
μ (mm^−1^)	4.80
Crystal size (mm)	0.36 × 0.06 × 0.04

Data collection	
Diffractometer	Rigaku Xcalibur Ruby Gemini ultra
Absorption correction	Analytical [*CrysAlis PRO* (Rigaku Oxford Diffraction, 2015 ▸), based on expressions derived by Clark & Reid (1995 ▸)]
*T*_min_, *T*_max_	0.499, 0.853
No. of measured, independent and observed [*I* > 2σ(*I*)] reflections	11115, 4314, 3371
*R*_int_	0.035
(sin θ/λ)_max_ (Å^−1^)	0.576

Refinement	
*R*[*F* ^2^ > 2σ(*F* ^2^)], *wR*(*F* ^2^), *S*	0.040, 0.106, 1.05
No. of reflections	4314
No. of parameters	516
No. of restraints	403
H-atom treatment	H atoms treated by a mixture of independent and constrained refinement
Δρ_max_, Δρ_min_ (e Å^−3^)	0.74, −0.72

Computer programs: *CrysAlis PRO* (Rigaku Oxford Diffraction, 2015 ▸), *SHELXT* (Sheldrick, 2015 ▸a), *SHELXL2014* (Sheldrick, 2015 ▸b), *ORTEP-3 for Windows* (Farrugia, 2012 ▸) and *Mercury* (Macrae *et al.*, 2008 ▸).

## supplementary crystallographic information

### Crystal data

**Table:**

C_21_H_12_Br_2_F_13_NO·2CH_4_O	*D*_x_ = 1.834 Mg m^−^^3^
*M**_r_* = 765.22	Melting point: 513 K
Monoclinic, *P*2_1_/*c*	Cu *K*α radiation, λ = 1.54184 Å
*a* = 22.2362 (7) Å	Cell parameters from 3207 reflections
*b* = 6.7922 (18) Å	θ = 6.8–62.6°
*c* = 18.9098 (5) Å	µ = 4.80 mm^−^^1^
β = 103.989 (3)°	*T* = 173 K
*V* = 2771.3 (7) Å^3^	Needle, red
*Z* = 4	0.36 × 0.06 × 0.04 mm
*F*(000) = 1504	

### Data collection

**Table:**

Rigaku Xcalibur Ruby Gemini ultra diffractometer	4314 independent reflections
Radiation source: fine-focus sealed X-ray tube, Enhance Ultra (Cu) X-ray Source	3371 reflections with *I* > 2σ(*I*)
Mirror monochromator	*R*_int_ = 0.035
Detector resolution: 10.3575 pixels mm^-1^	θ_max_ = 62.7°, θ_min_ = 4.1°
ω scans	*h* = −25→22
Absorption correction: analytical [CrysAlis PRO (Rigaku Oxford Diffraction, 2015), based on expressions derived by Clark & Reid (1995)]	*k* = −7→6
*T*_min_ = 0.499, *T*_max_ = 0.853	*l* = −14→21
11115 measured reflections	

### Refinement

**Table:**

Refinement on *F*^2^	403 restraints
Least-squares matrix: full	Hydrogen site location: mixed
*R*[*F*^2^ > 2σ(*F*^2^)] = 0.040	H atoms treated by a mixture of independent and constrained refinement
*wR*(*F*^2^) = 0.106	*w* = 1/[σ^2^(*F*_o_^2^) + (0.0459*P*)^2^ + 2.6693*P*] where *P* = (*F*_o_^2^ + 2*F*_c_^2^)/3
*S* = 1.05	(Δ/σ)_max_ = 0.002
4314 reflections	Δρ_max_ = 0.74 e Å^−^^3^
516 parameters	Δρ_min_ = −0.72 e Å^−^^3^

### Special details

**Table:**

Geometry. All esds (except the esd in the dihedral angle between two l.s. planes) are estimated using the full covariance matrix. The cell esds are taken into account individually in the estimation of esds in distances, angles and torsion angles; correlations between esds in cell parameters are only used when they are defined by crystal symmetry. An approximate (isotropic) treatment of cell esds is used for estimating esds involving l.s. planes.

### Fractional atomic coordinates and isotropic or equivalent isotropic displacement parameters (Å^2^)

**Table:**

	*x*	*y*	*z*	*U*_iso_*/*U*_eq_	Occ. (<1)
Br1	0.72887 (2)	0.71430 (7)	0.85956 (2)	0.03986 (15)	
Br2	0.58110 (2)	0.70255 (10)	1.06685 (3)	0.06123 (19)	
O1	0.69939 (13)	0.7051 (4)	1.00942 (15)	0.0413 (7)	
N1	0.30370 (15)	0.7317 (5)	0.55358 (18)	0.0355 (8)	
C1	0.35778 (19)	0.7194 (6)	0.5334 (2)	0.0384 (10)	
H1	0.3574	0.7177	0.4830	0.046*	
C2	0.41297 (19)	0.7094 (6)	0.5841 (2)	0.0373 (10)	
H2	0.4505	0.7012	0.5686	0.045*	
C3	0.41516 (19)	0.7111 (6)	0.6584 (2)	0.0349 (9)	
C4	0.35844 (19)	0.7177 (6)	0.6781 (2)	0.0360 (9)	
H4	0.3575	0.7151	0.7280	0.043*	
C5	0.30411 (19)	0.7278 (6)	0.6247 (2)	0.0366 (10)	
H5	0.2658	0.7322	0.6386	0.044*	
C6	0.47455 (19)	0.7076 (6)	0.7121 (2)	0.0373 (10)	
H6	0.5111	0.6998	0.6946	0.045*	
C7	0.48119 (19)	0.7145 (6)	0.7842 (2)	0.0375 (10)	
H7	0.4440	0.7199	0.8005	0.045*	
C8	0.53856 (19)	0.7149 (6)	0.8404 (2)	0.0347 (9)	
C9	0.59774 (18)	0.7163 (6)	0.8264 (2)	0.0336 (9)	
H9	0.6016	0.7186	0.7775	0.040*	
C10	0.64987 (18)	0.7145 (6)	0.8824 (2)	0.0321 (9)	
C11	0.65004 (18)	0.7098 (6)	0.9584 (2)	0.0343 (9)	
C12	0.58882 (19)	0.7105 (6)	0.9690 (2)	0.0371 (9)	
C13	0.53587 (18)	0.7137 (6)	0.9137 (2)	0.0377 (10)	
H13	0.4966	0.7152	0.9254	0.045*	
C14	0.24393 (19)	0.7506 (6)	0.4984 (2)	0.0390 (10)	
H14A	0.2518	0.7731	0.4497	0.047*	
H14B	0.2205	0.8648	0.5103	0.047*	
C15	0.20596 (19)	0.5630 (6)	0.4972 (2)	0.0389 (10)	
H15A	0.2305	0.4488	0.4876	0.047*	
H15B	0.1971	0.5439	0.5456	0.047*	
C16	0.14596 (17)	0.5710 (6)	0.4401 (2)	0.0390 (10)	
F1	0.11416 (12)	0.7347 (4)	0.44718 (17)	0.0615 (8)	
F2	0.15626 (12)	0.5751 (5)	0.37277 (13)	0.0621 (8)	
C17	0.1052 (6)	0.3886 (19)	0.4512 (11)	0.032 (3)	0.538 (7)
F3	0.0880 (11)	0.395 (3)	0.5138 (13)	0.046 (3)	0.538 (7)
F4	0.1392 (9)	0.227 (2)	0.4520 (9)	0.047 (3)	0.538 (7)
C18	0.0443 (6)	0.398 (2)	0.3879 (6)	0.049 (5)	0.538 (7)
F5	0.0088 (6)	0.553 (2)	0.3897 (9)	0.096 (5)	0.538 (7)
F6	0.0511 (6)	0.380 (2)	0.3204 (6)	0.114 (6)	0.538 (7)
C19	0.0003 (5)	0.2195 (14)	0.3942 (5)	0.0466 (13)	0.538 (7)
F7	−0.0127 (5)	0.2142 (17)	0.4591 (4)	0.086 (4)	0.538 (7)
F8	0.0246 (3)	0.0455 (11)	0.3845 (7)	0.109 (4)	0.538 (7)
C20	−0.0657 (5)	0.2196 (13)	0.3402 (6)	0.049 (3)	0.538 (7)
F9	−0.0608 (7)	0.227 (2)	0.2719 (6)	0.096 (5)	0.538 (7)
F10	−0.0975 (3)	0.3771 (10)	0.3515 (4)	0.095 (3)	0.538 (7)
C21	−0.1086 (5)	0.0383 (14)	0.3430 (6)	0.0659 (17)	0.538 (7)
F11	−0.0834 (3)	−0.1257 (10)	0.3271 (7)	0.126 (4)	0.538 (7)
F12	−0.1620 (5)	0.063 (2)	0.2950 (6)	0.093 (4)	0.538 (7)
F13	−0.1200 (5)	0.020 (2)	0.4076 (5)	0.114 (6)	0.538 (7)
C17A	0.1030 (7)	0.396 (2)	0.4358 (14)	0.032 (3)	0.462 (7)
F3A	0.1343 (11)	0.230 (3)	0.4336 (12)	0.081 (7)	0.462 (7)
F4A	0.0912 (14)	0.406 (4)	0.5014 (16)	0.074 (8)	0.462 (7)
C18A	0.0435 (6)	0.3502 (19)	0.3801 (6)	0.034 (4)	0.462 (7)
F5A	0.0231 (6)	0.5312 (18)	0.3621 (8)	0.063 (3)	0.462 (7)
F6A	0.0670 (6)	0.2830 (18)	0.3267 (6)	0.055 (3)	0.462 (7)
C19A	−0.0047 (6)	0.2143 (16)	0.3974 (5)	0.0466 (13)	0.462 (7)
F7A	−0.0318 (5)	0.3151 (16)	0.4410 (6)	0.080 (4)	0.462 (7)
F8A	0.0262 (4)	0.0682 (13)	0.4367 (5)	0.070 (3)	0.462 (7)
C20A	−0.0509 (5)	0.1407 (16)	0.3308 (6)	0.051 (4)	0.462 (7)
F9A	−0.0241 (4)	−0.0059 (14)	0.3042 (5)	0.104 (4)	0.462 (7)
F10A	−0.0640 (8)	0.282 (2)	0.2817 (9)	0.110 (7)	0.462 (7)
C21A	−0.1099 (5)	0.0648 (16)	0.3461 (7)	0.0659 (17)	0.462 (7)
F11A	−0.1445 (3)	0.2054 (15)	0.3637 (7)	0.121 (4)	0.462 (7)
F12A	−0.0958 (6)	−0.061 (2)	0.4003 (7)	0.108 (6)	0.462 (7)
F13A	−0.1435 (7)	−0.028 (2)	0.2889 (7)	0.099 (5)	0.462 (7)
O1S	0.65266 (18)	0.2293 (6)	1.1488 (2)	0.0597 (9)	
H1S	0.678 (2)	0.321 (7)	1.146 (4)	0.10 (2)*	
C1S	0.6945 (3)	0.0714 (8)	1.1694 (3)	0.0690 (15)	
H1S1	0.7365	0.1231	1.1877	0.104*	
H1S2	0.6933	−0.0130	1.1270	0.104*	
H1S3	0.6827	−0.0057	1.2077	0.104*	
O2S	0.72828 (18)	0.5293 (6)	1.1399 (2)	0.0699 (11)	
H2S	0.713 (3)	0.582 (9)	1.0994 (17)	0.09 (2)*	
C2S	0.7847 (3)	0.5858 (10)	1.1829 (3)	0.0746 (16)	
H2S1	0.7859	0.7296	1.1876	0.112*	
H2S2	0.8178	0.5422	1.1605	0.112*	
H2S3	0.7906	0.5261	1.2312	0.112*	

### Atomic displacement parameters (Å^2^)

**Table:**

	*U*^11^	*U*^22^	*U*^33^	*U*^12^	*U*^13^	*U*^23^
Br1	0.0307 (2)	0.0410 (3)	0.0472 (3)	−0.00156 (19)	0.00816 (18)	−0.0061 (2)
Br2	0.0379 (3)	0.1009 (5)	0.0449 (3)	−0.0003 (3)	0.0099 (2)	0.0052 (3)
O1	0.0285 (15)	0.0506 (18)	0.0395 (16)	−0.0031 (13)	−0.0018 (13)	0.0026 (13)
N1	0.0327 (18)	0.0273 (18)	0.041 (2)	−0.0018 (14)	−0.0013 (15)	0.0000 (14)
C1	0.036 (2)	0.036 (2)	0.043 (2)	−0.0035 (18)	0.0078 (19)	−0.0013 (18)
C2	0.031 (2)	0.031 (2)	0.048 (3)	−0.0014 (18)	0.0064 (18)	−0.0033 (19)
C3	0.033 (2)	0.0195 (19)	0.049 (3)	−0.0007 (17)	0.0035 (18)	−0.0019 (18)
C4	0.034 (2)	0.032 (2)	0.039 (2)	−0.0023 (18)	0.0025 (18)	0.0009 (18)
C5	0.033 (2)	0.031 (2)	0.045 (3)	−0.0029 (17)	0.0076 (18)	−0.0012 (18)
C6	0.029 (2)	0.031 (2)	0.049 (3)	−0.0020 (17)	0.0040 (18)	−0.0021 (19)
C7	0.031 (2)	0.029 (2)	0.049 (3)	−0.0009 (18)	0.0020 (18)	0.0005 (19)
C8	0.032 (2)	0.023 (2)	0.044 (2)	−0.0009 (17)	−0.0006 (18)	0.0005 (17)
C9	0.033 (2)	0.025 (2)	0.040 (2)	0.0000 (17)	0.0032 (17)	−0.0034 (17)
C10	0.032 (2)	0.0229 (19)	0.040 (2)	−0.0025 (16)	0.0058 (17)	0.0009 (17)
C11	0.031 (2)	0.024 (2)	0.046 (2)	−0.0028 (17)	0.0053 (19)	−0.0001 (18)
C12	0.032 (2)	0.036 (2)	0.041 (2)	−0.0006 (18)	0.0045 (18)	0.0013 (18)
C13	0.026 (2)	0.033 (2)	0.052 (3)	−0.0002 (17)	0.0058 (18)	−0.0008 (19)
C14	0.031 (2)	0.040 (3)	0.040 (2)	0.0012 (18)	−0.0048 (18)	0.0039 (18)
C15	0.039 (2)	0.035 (2)	0.038 (2)	0.0002 (19)	−0.0012 (18)	0.0013 (18)
C16	0.031 (2)	0.041 (3)	0.042 (2)	0.0029 (19)	0.0038 (18)	0.0035 (19)
F1	0.0363 (14)	0.0402 (15)	0.097 (2)	0.0058 (12)	−0.0057 (14)	−0.0023 (14)
F2	0.0504 (16)	0.094 (2)	0.0383 (15)	−0.0251 (15)	0.0026 (12)	0.0056 (14)
C17	0.037 (3)	0.045 (3)	0.014 (8)	−0.001 (2)	0.006 (3)	0.000 (3)
F3	0.057 (5)	0.061 (6)	0.026 (6)	−0.017 (5)	0.018 (4)	−0.006 (4)
F4	0.045 (5)	0.030 (5)	0.056 (5)	−0.005 (3)	−0.006 (3)	0.002 (3)
C18	0.049 (9)	0.059 (11)	0.041 (8)	−0.031 (7)	0.017 (6)	−0.022 (7)
F5	0.034 (5)	0.049 (4)	0.185 (15)	0.003 (3)	−0.011 (6)	0.010 (7)
F6	0.075 (9)	0.211 (18)	0.047 (5)	−0.084 (10)	−0.002 (5)	0.002 (9)
C19	0.036 (3)	0.051 (3)	0.052 (3)	−0.009 (2)	0.010 (2)	0.000 (2)
F7	0.071 (7)	0.129 (10)	0.050 (4)	−0.052 (7)	−0.001 (4)	0.020 (5)
F8	0.049 (4)	0.054 (4)	0.208 (11)	0.002 (3)	−0.002 (6)	−0.039 (6)
C20	0.050 (7)	0.054 (9)	0.042 (6)	−0.012 (6)	0.010 (5)	−0.020 (7)
F9	0.093 (8)	0.165 (11)	0.032 (6)	−0.081 (8)	0.019 (5)	−0.020 (6)
F10	0.045 (4)	0.081 (5)	0.140 (7)	0.006 (3)	−0.016 (4)	−0.024 (4)
C21	0.047 (3)	0.084 (5)	0.062 (4)	−0.028 (3)	0.006 (3)	−0.003 (4)
F11	0.088 (6)	0.070 (5)	0.220 (12)	−0.031 (4)	0.036 (7)	−0.028 (6)
F12	0.058 (7)	0.122 (11)	0.086 (6)	−0.041 (6)	−0.008 (4)	−0.004 (6)
F13	0.067 (8)	0.211 (17)	0.063 (5)	−0.073 (9)	0.015 (5)	0.000 (7)
C17A	0.037 (3)	0.045 (3)	0.014 (8)	−0.001 (2)	0.006 (3)	0.000 (3)
F3A	0.044 (7)	0.064 (9)	0.131 (18)	0.006 (6)	0.009 (10)	−0.042 (9)
F4A	0.068 (8)	0.116 (12)	0.034 (11)	−0.049 (7)	0.006 (7)	0.004 (6)
C18A	0.046 (9)	0.022 (6)	0.023 (8)	−0.004 (5)	−0.015 (6)	−0.003 (5)
F5A	0.033 (7)	0.052 (7)	0.085 (8)	0.002 (5)	−0.019 (5)	0.015 (5)
F6A	0.042 (5)	0.094 (7)	0.032 (5)	−0.014 (4)	0.014 (4)	−0.017 (4)
C19A	0.036 (3)	0.051 (3)	0.052 (3)	−0.009 (2)	0.010 (2)	0.000 (2)
F7A	0.063 (6)	0.106 (9)	0.087 (8)	−0.035 (5)	0.049 (6)	−0.060 (6)
F8A	0.055 (5)	0.058 (5)	0.084 (6)	−0.024 (4)	−0.010 (5)	0.016 (5)
C20A	0.057 (9)	0.060 (10)	0.035 (8)	−0.020 (7)	0.006 (6)	−0.026 (7)
F9A	0.077 (5)	0.139 (9)	0.103 (6)	−0.033 (6)	0.032 (5)	−0.074 (6)
F10A	0.070 (9)	0.135 (11)	0.091 (13)	−0.054 (7)	−0.048 (7)	0.055 (9)
C21A	0.047 (3)	0.084 (5)	0.062 (4)	−0.028 (3)	0.006 (3)	−0.003 (4)
F11A	0.037 (4)	0.149 (9)	0.178 (11)	−0.014 (5)	0.029 (5)	−0.058 (8)
F12A	0.066 (9)	0.145 (12)	0.100 (10)	−0.057 (8)	−0.005 (6)	0.049 (9)
F13A	0.075 (11)	0.140 (14)	0.073 (6)	−0.058 (8)	−0.002 (6)	−0.022 (7)
O1S	0.059 (2)	0.061 (2)	0.060 (2)	−0.001 (2)	0.0160 (18)	0.0056 (18)
C1S	0.075 (4)	0.058 (3)	0.072 (4)	0.006 (3)	0.012 (3)	0.010 (3)
O2S	0.065 (2)	0.070 (3)	0.063 (2)	−0.013 (2)	−0.0062 (19)	0.023 (2)
C2S	0.065 (4)	0.086 (4)	0.065 (4)	−0.006 (3)	−0.001 (3)	0.008 (3)

### Geometric parameters (Å, º)

**Table:**

Br1—C10	1.908 (4)	C17—C18	1.576 (9)
Br2—C12	1.900 (4)	C18—F6	1.325 (7)
O1—C11	1.274 (5)	C18—F5	1.326 (7)
N1—C5	1.344 (5)	C18—C19	1.579 (9)
N1—C1	1.350 (5)	C19—F7	1.328 (7)
N1—C14	1.484 (5)	C19—F8	1.331 (7)
C1—C2	1.365 (6)	C19—C20	1.573 (9)
C1—H1	0.9500	C20—F9	1.323 (7)
C2—C3	1.393 (6)	C20—F10	1.327 (7)
C2—H2	0.9500	C20—C21	1.566 (9)
C3—C4	1.400 (6)	C21—F13	1.313 (7)
C3—C6	1.459 (5)	C21—F11	1.314 (7)
C4—C5	1.375 (6)	C21—F12	1.319 (7)
C4—H4	0.9500	C17A—F3A	1.331 (7)
C5—H5	0.9500	C17A—F4A	1.331 (7)
C6—C7	1.337 (6)	C17A—C18A	1.513 (8)
C6—H6	0.9500	C18A—F6A	1.325 (7)
C7—C8	1.450 (5)	C18A—F5A	1.326 (7)
C7—H7	0.9500	C18A—C19A	1.509 (9)
C8—C13	1.401 (6)	C19A—F7A	1.322 (7)
C8—C9	1.404 (6)	C19A—F8A	1.328 (7)
C9—C10	1.368 (5)	C19A—C20A	1.506 (9)
C9—H9	0.9500	C20A—F10A	1.318 (7)
C10—C11	1.436 (6)	C20A—F9A	1.321 (7)
C11—C12	1.423 (6)	C20A—C21A	1.500 (9)
C12—C13	1.373 (6)	C21A—F12A	1.314 (7)
C13—H13	0.9500	C21A—F11A	1.319 (7)
C14—C15	1.526 (6)	C21A—F13A	1.319 (7)
C14—H14A	0.9900	O1S—C1S	1.411 (6)
C14—H14B	0.9900	O1S—H1S	0.845 (11)
C15—C16	1.501 (5)	C1S—H1S1	0.9800
C15—H15A	0.9900	C1S—H1S2	0.9800
C15—H15B	0.9900	C1S—H1S3	0.9800
C16—F1	1.342 (4)	O2S—C2S	1.374 (6)
C16—F2	1.348 (4)	O2S—H2S	0.838 (11)
C16—C17A	1.515 (8)	C2S—H2S1	0.9800
C16—C17	1.578 (9)	C2S—H2S2	0.9800
C17—F4	1.329 (6)	C2S—H2S3	0.9800
C17—F3	1.330 (6)		
			
C5—N1—C1	119.4 (3)	F6—C18—F5	107.6 (7)
C5—N1—C14	119.6 (4)	F6—C18—C17	116.8 (14)
C1—N1—C14	120.9 (4)	F5—C18—C17	114.8 (13)
N1—C1—C2	121.0 (4)	F6—C18—C19	102.9 (11)
N1—C1—H1	119.5	F5—C18—C19	102.9 (10)
C2—C1—H1	119.5	C17—C18—C19	110.3 (11)
C1—C2—C3	121.0 (4)	F7—C19—F8	106.9 (7)
C1—C2—H2	119.5	F7—C19—C20	102.9 (9)
C3—C2—H2	119.5	F8—C19—C20	104.9 (8)
C2—C3—C4	117.0 (4)	F7—C19—C18	111.6 (9)
C2—C3—C6	120.5 (4)	F8—C19—C18	112.9 (9)
C4—C3—C6	122.5 (4)	C20—C19—C18	116.7 (9)
C5—C4—C3	119.7 (4)	F9—C20—F10	107.3 (7)
C5—C4—H4	120.2	F9—C20—C21	105.0 (10)
C3—C4—H4	120.2	F10—C20—C21	106.0 (7)
N1—C5—C4	121.8 (4)	F9—C20—C19	110.5 (10)
N1—C5—H5	119.1	F10—C20—C19	110.2 (7)
C4—C5—H5	119.1	C21—C20—C19	117.2 (9)
C7—C6—C3	124.6 (4)	F13—C21—F11	108.8 (7)
C7—C6—H6	117.7	F13—C21—F12	107.8 (7)
C3—C6—H6	117.7	F11—C21—F12	108.2 (7)
C6—C7—C8	127.5 (4)	F13—C21—C20	111.3 (9)
C6—C7—H7	116.3	F11—C21—C20	111.3 (7)
C8—C7—H7	116.3	F12—C21—C20	109.4 (10)
C13—C8—C9	116.9 (4)	F3A—C17A—F4A	107.1 (7)
C13—C8—C7	119.0 (4)	F3A—C17A—C18A	100.4 (15)
C9—C8—C7	124.1 (4)	F4A—C17A—C18A	108.8 (18)
C10—C9—C8	120.7 (4)	F3A—C17A—C16	109.8 (16)
C10—C9—H9	119.6	F4A—C17A—C16	100 (2)
C8—C9—H9	119.6	C18A—C17A—C16	129.3 (15)
C9—C10—C11	124.9 (4)	F6A—C18A—F5A	107.3 (7)
C9—C10—Br1	118.6 (3)	F6A—C18A—C19A	112.8 (11)
C11—C10—Br1	116.5 (3)	F5A—C18A—C19A	114.0 (11)
O1—C11—C12	124.8 (4)	F6A—C18A—C17A	99.2 (15)
O1—C11—C10	123.5 (4)	F5A—C18A—C17A	100.1 (13)
C12—C11—C10	111.7 (3)	C19A—C18A—C17A	121.5 (13)
C13—C12—C11	124.5 (4)	F7A—C19A—F8A	106.9 (7)
C13—C12—Br2	118.6 (3)	F7A—C19A—C20A	111.8 (9)
C11—C12—Br2	116.9 (3)	F8A—C19A—C20A	112.1 (10)
C12—C13—C8	121.3 (4)	F7A—C19A—C18A	105.9 (10)
C12—C13—H13	119.4	F8A—C19A—C18A	106.3 (9)
C8—C13—H13	119.4	C20A—C19A—C18A	113.4 (10)
N1—C14—C15	109.6 (3)	F10A—C20A—F9A	108.9 (7)
N1—C14—H14A	109.7	F10A—C20A—C21A	109.4 (13)
C15—C14—H14A	109.7	F9A—C20A—C21A	108.1 (8)
N1—C14—H14B	109.7	F10A—C20A—C19A	109.7 (11)
C15—C14—H14B	109.7	F9A—C20A—C19A	106.8 (9)
H14A—C14—H14B	108.2	C21A—C20A—C19A	113.8 (10)
C16—C15—C14	111.9 (3)	F12A—C21A—F11A	108.6 (7)
C16—C15—H15A	109.2	F12A—C21A—F13A	107.9 (7)
C14—C15—H15A	109.2	F11A—C21A—F13A	107.7 (7)
C16—C15—H15B	109.2	F12A—C21A—C20A	108.7 (10)
C14—C15—H15B	109.2	F11A—C21A—C20A	113.0 (9)
H15A—C15—H15B	107.9	F13A—C21A—C20A	110.8 (11)
F1—C16—F2	106.9 (3)	C1S—O1S—H1S	100 (5)
F1—C16—C15	110.9 (3)	O1S—C1S—H1S1	109.5
F2—C16—C15	110.9 (3)	O1S—C1S—H1S2	109.5
F1—C16—C17A	108.4 (9)	H1S1—C1S—H1S2	109.5
F2—C16—C17A	102.4 (11)	O1S—C1S—H1S3	109.5
C15—C16—C17A	116.7 (8)	H1S1—C1S—H1S3	109.5
F1—C16—C17	107.7 (8)	H1S2—C1S—H1S3	109.5
F2—C16—C17	112.0 (9)	C2S—O2S—H2S	121 (5)
C15—C16—C17	108.4 (6)	O2S—C2S—H2S1	109.5
F4—C17—F3	107.5 (6)	O2S—C2S—H2S2	109.5
F4—C17—C18	114.9 (14)	H2S1—C2S—H2S2	109.5
F3—C17—C18	107.2 (15)	O2S—C2S—H2S3	109.5
F4—C17—C16	107.8 (12)	H2S1—C2S—H2S3	109.5
F3—C17—C16	113.0 (17)	H2S2—C2S—H2S3	109.5
C18—C17—C16	106.5 (12)		

### Hydrogen-bond geometry (Å, º)

**Table:**

*D*—H···*A*	*D*—H	H···*A*	*D*···*A*	*D*—H···*A*
O1*S*—H1*S*···O2*S*	0.85 (1)	1.83 (1)	2.674 (5)	176 (7)
O2*S*—H2*S*···O1	0.84 (1)	1.85 (2)	2.675 (4)	167 (6)
